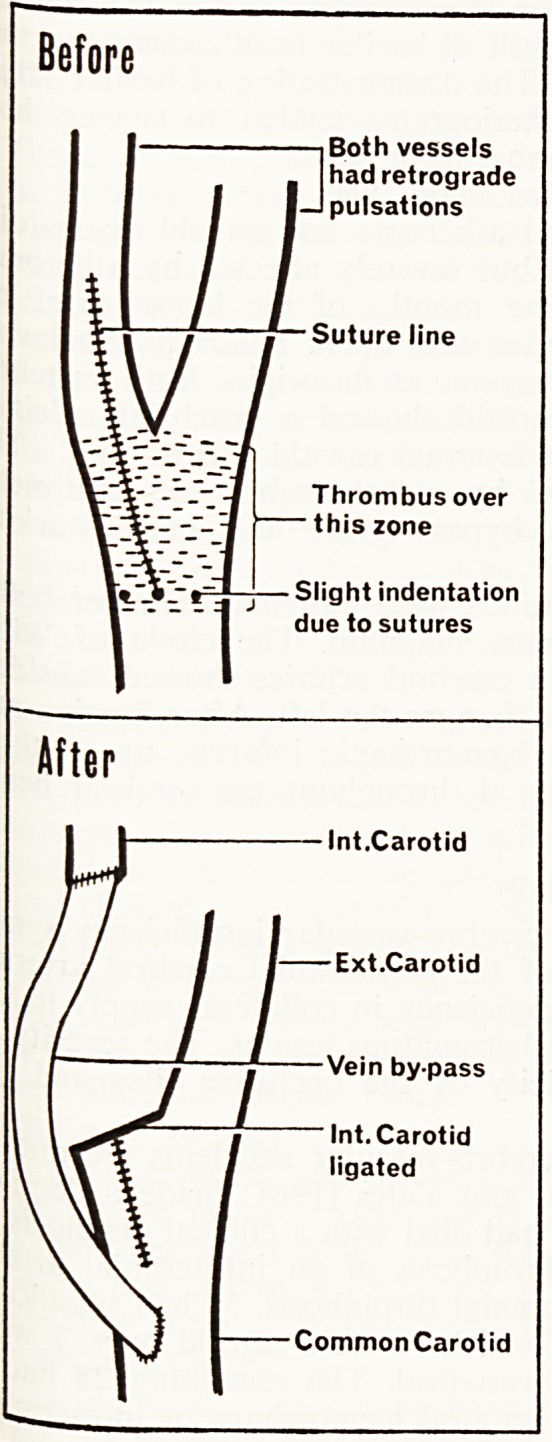# Case Reports

**Published:** 1967-07

**Authors:** A. A. J. Barros D'Sa


### CASE REPORTS

A. A. J. BARROS D'SA

*Medical Student*

*The Leonard-Pritchard-Tibbits Prize of the University of Bristol requires that an undergraduate should " submit six case histories of surgical patients under his care during the term of his appointment as dresser with a brief commentary on each ". The reports that follow are taken from the winning essay for* 1966-67; *they form a commentary upon arterial surgery as seen by a contemporary student—Editors.*

### Case 1

This is the case of a pensioner aged seventy-six years. She was first seen in the out-patients department by Mr. Peacock on the 15th July, 1965. She was then seventy-five years old. Her general practitioner had diagnosed an abdominal aortic aneurysm. On examination it was felt that she could stand having it resected. However, the patient was reluctant and in view of her age she was not pressed to undergo the operation. The question of rupture was explained to her. If ever she had much pain she would be gladly seen again.

Almost a year later the patient was admitted as an emergency at the Bristol Royal Infirmary. She had been seen by her doctor that morning with a three-day history of a great deal of pain in the abdomen. On examination he had found her pale, her pulse was 100/min., regular and of fair volume, her B.P. 160/90 and her heart sounds I and II heard in all areas. There was a large pulsating mass in the lumbar and umbilical regions of her abdomen and a diagnosis of a rupturing aneurysm was made.

On admission to hospital she gave a history of a dull ache in the abdomen and back radiating down to the buttocks. This had lasted ten months and had suddenly got worse three days previously. The pain was mild in the back and spasmodic in the buttocks. She could only walk a distance of a hundred yards. There was no history of breathlessness or headaches.

*Previous medical history:* Gastroenterostomy before 1955. Eczema treated and cleared in 1955. Radical mastectomy for scirrhous carcinoma of the left breast in 1955. Early Dupuytren's contracture of both palms in 1962.

*On examination:* The patient was a small, frail looking lady with a pale complexion. The hair was thin and fine. The hands, fingers and finger nails were normal. Her temperature was 97.5°F.

The pulse was 90 per minute, regular and of normal volume and form. Both radial pulses were felt and were of equal volume. The jugular venous pressure was not raised. The blood pressure was 190/90mm. mercury. On the left chest was the scar of the radical mastectomy. The apex beat was not visible but on palpation was localised to the fifth intercostal space, four inches from the mid-sternal line. There was no thrill. The first and second heart sounds were audible and normal in all areas. There were no murmurs or any adventitious sounds. The vessels of the fundi of the eyes were normal.

An abdominal scar of a right paramedian incision was present. In the region of the umbilicus there was an oval swelling. This was found to be a pulsatile mass about four inches by six inches. It was tender to the touch and the pulsations were expansile.

Both the femoral pulses were of equal and good volume. The posterior tibial and dorsalis pedis arteries were palpable in both limbs and were of equal and good volume on both sides. The two carotids were palpable in the neck and were of equal volume. Both the lower limbs were warm and there was no difference in temperature between them.

Examination of the respiratory and nervous systems revealed no abnormality.

The diagnosis of a leaking aneurysm was made. She was sedated with $\frac{1}{3}$ grain of omnopon intramuscularly.

*Investigations:* An x-ray of the chest on 7th May, 1966, showed some unfolding of the aorta with calcification. No lung lesion was seen.

An x-ray of the abdomen on the same date showed the intestinal gas pattern to be normal. There was a line of calcification to the left of the lumbar spine which was in the wall of the abdominal aorta. The appearance was consistent with an aueurysm (Plate XXVII).

Liver function tests produced a total bilirubin of 0.2mg%; serum proteins on electrophoresis were within normal limits. The hæmoglobin was 12.0G%, the packed cell volume 35%, the M.C.H.C. 34% and the E.S.R. 19mm. per hour. E.C.G. showed evidence of ischæmic heart disease.

On plain x-ray films of the urinary tract the renal outlines were within normal limits. The calcification in the wall of the abdominal aortic aneurysm was obvious. Degenerative changes were seen in the lumbar spine. On excretion urography no abnormality of the urinary tract was shown.

Blood urea and electrolyte levels were done. Urea was 26mg/100 ml, plasma sodium 141mEq/litre, plasma potassium 3.8mEq/litre, plasma chloride 106mEq/litre, plasma bicarbonate 26.5mEq/litre, plasma protein 6.3%.

She remained quite comfortable during the first few days and slept well, being given 20 grains of dichloralphenazone every night.

On the 12th May she vomited and complained of abdominal pain. By the 16th May she had back pain as well. It was decided that she should have her aneurysm explored with a view to resection and grafting. On the 17th May the abdominal aortic aneurysm was explored by Mr. Terblanche. A longitudinal left paramedian incision was made. A resectable aneurysm was found in the aorta below the origin of the renal arteries and below the level of the left renal vein and extending up to the bifurcation and into the common iliac arteries. During the early part of the anæsthetic, which was administered by Professor Clutton-Brock, the patient developed a marked bundle-branch block. This necessitated the use of isoprenaline. Her general condition was unsatisfactory and it was decided that she would not stand resection and grafting of her aneurysm. Instead, however, the aneurysm was wired, using a Peacock Stainless Steel Wiring machine inserting about two hundred feet of stainless steel wire from three sites (Plate XXVIII). Reconstitution of the anterior abdominal wall was performed in layers with nylon sutures, and silk was used for the skin.

Antibiotic therapy was begun with one mega-unit of penicillin and one gram of streptomycin. This was continued with half a mega-unit of penicillin six hourly and half a gram of streptomycin twelve hourly for seven days post-operatively. She was put into the intensive care unit and had to have constant supervision, and isoprenaline to treat her heart block. She subsequently returned to the ward and her further convalescence over the next few days was slow.

She received intravenous fluids; one fifth normal dextrose saline, supplemented as necessary with potassium. Oral fluids were limited. However, she continued to have very large quantities of gastric aspirate amounting on one day to almost three litres.

An x-ray of the abdomen (supine and erect) on the 25th May displayed no evidence of intestinal obstruction or ileus. A barium meal showed the stomach to be a little atonic and to contain a considerable amount of resting juice but no lesion was seen in it or in the duodenum. Barium passed freely into the jejunum but no further. The aspiration tube had passed into the second part of the duodenum and had looped itself, so that the tip was still in the stomach. Obstruction in the upper jejunum was suspected and it was decided to re-explore the abdomen.

On the 27th May, 1966, laparotomy was performed by Mr. Terblanche. Professor Clutton-Brock again administered the anæsthetic. There was no cardiac trouble this time. The obstruction was found to be at the site of her gastro-enterostomy efferent loop which had become kinked by adhesions to the aneurysm. The wall of the aneurysm had become fleshy and œdematous, probably as a result of the wiring and subsequent thrombosis that had taken place. The aneurysm at this stage was no longer pulsatile.

Her subsequent post-operative course was satisfactory and there was no longer any gastric aspirate. A slight urinary infection was cleared up with a course of sulphadimidine and she was eventually transferred to convalescence on the 20th June, 1966. She did have persistent lower back pain and a neurogenic type of pain down the inner side of her left thigh, but this was much better by the time of her discharge.

Three weeks later on the 11th July she was readmitted from convalescence at Axbridge because of persistent diarrhœa, associated with poor appetite and occasional vomiting. On examination she was dehydrated. Her tongue was dry and her skin was lax. The pulse was 80 per minute, regular and of normal volume. The blood pressure was 120/90mm. Hg. There was no œdema, her jugular venous pressure was not raised and her peripheral pulses were normal and equal on both sides.

The scar of the operation was well healed. There was tenderness over the pulsatile aortic aneurysm especially in the left hypochondrium. There were no other masses and the bowel sounds were normal. Examination *per rectum* showed soft greenish fæces but nothing else. Her respiratory and central nervous systems revealed no abnormality. After she had been rehydrated her hæmoglobin was 12G%, her electrolytes were normal and her stool cultures negative. Bilirubin and serum proteins were within normal limits. No real cause for her diarrhœa was found. She was treated with codeine phosphate, chlorpromazine, iron and vitamin C and her general condition improved considerably. She was discharged on the 29th July, 1966, to live with a nephew in Derbyshire. She was quite happy to go to live with him. She will be seen in out patients when she is next in this region.

### DISCUSSION

Before the advent of successful reconstructive aortic surgery, abdominal aortic aneurysm was a condition for which there was no effective therapy. In 1950 Estes reviewed 102 cases of abdominal aortic aneurysms and made an effort to outline clearly the natural history of this disease. Since then excision of aneurysms with replacements by homografts and more recently by synthetic materials became widespread.

Pain is the most important symptom of a rupturing abdominal aneurysm. This is accompanied by abdominal discomfort. Significant signs in the diagnosis of an unruptured abdominal aneurysm include the palpation of a pulsatile, expansile abdominal mass, and curvilinear calcification on abdominal radiography. Intravenous aortagraphy, though used to establish the diagnosis by a few surgeons in the United States, is considered an unnecessary investigation by most vascular surgeons today. An abdominal aneurysm can be easily diagnosed without the aid of an aortogram. Also when the aorta, which may be thinned and contains atheromatous plaques, is punctured in translumbar aortography, there is a danger of rupturing it or dislodging a plaque or thrombus. The differential diagnosis of an abdominal aneurysm includes a pulsatile normal aorta in thin patients or patients with lordotic spines, abdominal masses adjacent to the abdominal aorta, and tortuosity of a normal-sized aorta.

The patient was known to have the abdominal aneurysm almost a year before she was admitted to hospital. Although she was not pressed to have the operation, because of her reluctance, she was undergoing a great risk of rupture. Operation after rupture has occurred leads to a much higher mortality than operation before rupture. De Bakey showed this very clearly in the following figures (De Bakey, 1954) :—

| | Total operations | Mortality |
|---|---|---|
| Non-ruptured aorta ......... | 142 | 10% |
| Ruptured aorta .............. | 22 | 40% |

When a rupture occurs it may be into the retroperitoneum, the peritoneal cavity, or the duodenum. Rupture is followed by collapse, sudden abdominal pain, rigidity and ileus. The outlook for patients who suffer from untreated aneurysm of the abdominal aorta is poor. A great number die within a year of diagnosis and most are dead within five years.

The patient's blood pressure was 160/90 when examined by the G.P., and 190/90 on admission to hospital. The incidence of hypertension in abdominal aortic aneurysms is well known. Out of 94 patients with abdominal aneurysms, Estes (1958) found that twenty-five of the sixty-eight males (36.8%) and sixteen of the twenty-six females were hypertensive. For the purpose of analysis hypertension was considered to be present if the blood pressure was above 160 systolic or 100 diastolic, expressed in millimetres of mercury. Steinberg and Stein (1966) also produced a similar percentage when they found that 80 of their 200 consecutive cases (40%) were hypertensive.

The patient's aneurysm extended from below the level of the renal arteries to the bifurcation of the aorta into the common iliac arteries. This is the common site for an abdominal aortic aneurysm. Of 200 consecutive cases of aortic aneurysms diagnosed by intravenous aortography Steinberg and Stein found 12 patients (6%) with aneurysms above the level of the renal arteries. In 36 (18%) the aneurysm was primarily at the bifurcation of the aorta. In almost 95% of the cases the aneurysm was below the renal arteries.

While many thoracic aneurysms used to be syphilitic in origin the greater percentage of abdominal aneurysms are atherosclerotic in origin, usually involving the lower abdominal aorta as already mentioned. The reason for this anatomic predilection has never been completely clear but the explanation of Blakemore deserves careful consideration. He suggested that the explanation of the development of aneurysm resulting from atherosclerosis of the lower abdominal aorta was due to several factors:—

(a) Widespread atherosclerosis tends to involve the entire aorta, including the abdominal segment;

(b) the pressure of pulse waves striking the aortic bifurcation and the iliac arteries tends to produce a reverse wave that meets the oncoming next pulse wave. This results in a stress on the aortic wall which is not well supported by sheathing or surrounding tissues at this point;

(c) the stress may be aggravated by the fact that the aorta is fixed at the diaphragm and by the iliac fossa. Between these two fixed points the aorta tends to elongate with therosclerosis. It usually deviates to the left. (This occurred in the patient, as seen on the x-ray.) This bending tube tends with further strain to dilate into a fusiform aneurysm. This lack of fixation and the deviation forward also explains why erosion of the spine is rare in abdominal aneurysms as compared with thoracic aneurysms (Blakemore and Voorhees, 1954).

Because of the poor general condition of the patient during the operation, wiring of the aneurysm was performed in place of the favoured procedure today of resection and replacement. The replacement can be with a homograft or with a prosthesis of Teflon Dacron, Orlon, Vinyon N or other plastic. Other types of operation, some only occasionally performed now, include partial or complete external reinforcement or wrapping with cellophane and other materials. The purpose of wiring of the aneurysm is to produce thrombosis within the aneurysm, with or without endothermy.

What could one say of the prognosis of this patient? It would be necessary here to consider several factors. By reason of her age she cannot expect to live very long. The state of her heart is also a factor against her. However, had she not had surgical intervention her prognosis would have been even poorer.

### REFERENCES

(1) Aird I (1958): Companion in Surgical Studies.

(2) Blakemore, A. H., and Voorhees, A. R., JR (1954): Aneurysm of the Aorta; a review of 365 cases. Angiology **5**, 209.

(3) De Bakey, M. E., and Cooley, D. A. (1954): Treatment of Aneurysms of the Aorta by resection and restoration of continuity with aortic homograft. Angiology **5**, 251.

(4) Estes, J. E. (1950): Abdominal Aortic Aneurysm: Study of 102 Cases. Circulation **2**, 258-264.

(5) Schatz, I. J., Fairbairn, J. F., and Juergens, J. L. (1962): Abdominal Aortic Aneurysms: A Reappraisal. Circulation **26**, 200-205.

(6) Steinberg, I., and Moore, S. W. (1961): Intravenous Abdominal Aortography in Treatment of Abdominal Aneurysms. J.A.M.A. **175**, 446-451.

(7) Steinberg, I., and Stein, H. L. (1966) : Arteriosclerotic Abdominal Aneurysms. J.A.M.A. **195,** 1025-1029.

(8) Wright, I S, Undaneta, E, and Wright, B (1956): Reopening the Case of the Abdominal Aortic aneurysm. Circulation **13**, 754-768.

### Case 4

This patient was first seen by Mr. Peacock at an out-patients clinic in August 1964. He was an insurance superintendent and was then 57 years old. He had a history of four to five years of attacks of a grey film descending over his left eye, partially or completely blocking his sight. The attacks had been infrequent at first, having come on every six months or so, but were now occurring more often and in the previous week he had had two very brief attacks. Two months previously in June, 1964, he had had an epileptiform convulsion lasting five to ten minutes during his sleep. At the time he was lying on his left side and it was suggested that there might have been occlusion of his left internal carotid artery. His blood pressure after the attack was found by his general practitioner to be 80/50 mm. Hg.

Investigations carried out at the Burden Neurological Institute had shown (1) *E.E.G.:* an irregular " delta focus " in the left emporal region. (2) *Left carotid angiogram:* Severe carotid stenosis 2 cms. beyond the origin of the internal carotid extending over a distance of 5 cms., with retadation of the intracranial circulation rate. The appearances were those of severe atheroma. There was an excellent collateral supply through the occipital artery to the basilar.

Besides the atheromatous block mentioned above it was felt that there might possibly have been a stenosis of the innominate artery as there was a systolic bruit over the right carotid, and the blood pressure in the right arm was 90/60 mm. Hg., whereas on the left side it was 120/80 mm. Hg. It was felt advisable to do an angiogram on the innominate artery by retrograde catheterisation of his femoral artery so that a complete picture of the vascular supply to the brain could be obtained. It also seemed advisable to do a carotid endarterectomy on the left side.

The patient was admitted to the Bristol Royal Infirmary on the 15th September, 1964. He had no history of hypertension, tuberculosis or diabetes. He had smoked twenty cigarettes a day for many years. A review of his systems revealed nothing more than was already known.

*On examination* he was found to be a fit looking middle-aged man. There was no clubbing of fingers, cyanosis or jaundice. There were no signs of anæmia or dehydration.

His pulse was 80/minute and regular. The left radial pulse was a greater volume than the right. The radial pulse was delayed but of normal character. The vessel wall was not palpable. The jugular venous pulse was not raised. The apex beat was not palpable. On auscultation the first and second heart sounds were heard but were reduced in the aortic area. There were no

Plate XXVII

The above x-ray of the abdomen taken before the operation shows the curvilinear calcification in the wall of the abdominal aorta. This is seen to the left of the spine with its convexity towards the left. This is the usual direction of deviation of an abdominal aneurysm.

Plate XXVIII

This is an x-ray print of the aneurysm showing the steel wiring in the aneurysm. When the aneurysm was seen at the second operation ten days after the wiring it was no longer pulsatile.

murmurs. There was a systolic thrill and bruit over the right carotid. All the peripheral pulses were found to be normal. The blood pressure was 80/50 mm. Hg in the right arm and 130/80 mm. Hg in the left arm. The temperature was 98.2°F.

The trachea was central and there were no palpable neck glands. The chest was barrel-shaped and had a fair degree of expansion, this being greater on the right than on the left. The percussion note was resonant and the breath sounds were vesicular. There were no adventitious sounds. Examination of the central nervous system and the abdomen revealed no abnormality. The fundi were normal.

*Investigations*: His hæmoglobin was 99%; packed cell volume 43%; erythrocyte sedimentation rate 12 mm/hr., serum cholesterol 215 mg/100 ml., which was within normal limits; Wassermann, Cardiolipin W.R., Reiter Protein Complement Fixation, and Price's Precipitin Reaction tests were all negative.

Aortography was carried out by the introduction of a catheter into the right femoral artery by the Seldinger technique. The catheter was passed upwards so that its tip lay in the ascending aorta. An injection of 30 c.c. of 85% "Hypaque" was given. Films were taken at the rate of four a second for three seconds. The arch of the aorta appeared normal and the origin of the major vessels appeared normal. The right subclavian artery appeared to be blocked just beyond its origin and the left vertebral artery was also blocked. Atheromatous changes were seen in both the common carotid arteries.

On the 26th September, 1964, Mr. Peacock explored the left internal carotid artery. This was done under hypothermia using a left submandibular incision. A very adherent mass of glands was found, making it difficult to mobilise the artery. However when the artery was exposed it was found to be normal at the beginning of the internal carotid, although there was atheroma present at the bifurcation. As a good pulse was felt in it it was decided not to re-explore the artery further, and it was thought that the narrowing was probably caused by pressure from without.

The patient developed some wound swelling post-operatively, but this settled on conservative treatment and he was sent home on the 2nd October, 1964.

When seen at a post-operative follow-up clinic a month later he was quite well generally except for a complaint of paræsthesia of his right hand. It was not quite clear whether he had early wasting of the thenar eminence of this hand.

Two months later in January, 1965, he had no symptoms at all. He was right handed and could play golf. His carotids were equally palpable on each side. There was a loud bruit over the right carotid and a softer one over the left. Examination of his central nervous system showed no abnormality.

When seen again in the follow up clinic six months later in July, 1965, he was well but complained of a pain in the corner of the left eye. He had not had a pain of this sort since the operation ten months previously.

In January, 1966, there was a recurrence of visual attacks, these being in the right eye. They occurred about once a week, and each attack lasted a few minutes, causing a partial blindness. (Before 1964 when the patient had had attacks in his left eye they had caused complete blindness.) There was also a loss of light touch in the 3rd-5th fingers of the left hand.

He was admitted to the Bristol Royal Infirmary on the 6th April, 1966, for two days; again on the 29th April for three days and finally on the 13th May, 1966. On examination he was found to be relatively fit. The blood pressure in the left arm was 120/80 mm. Hg, but was unobtainable in the right. No pulses were palpable in the right arm. All the pulses were normal in the left arm. The right carotid was weaker than the left. There was a bilateral carotid bruit, but more marked on the right. Both femoral pulses were palpable, but the pulses distal to these were not palpable.

*Diagnosis*: Clinically the patient was thought to have generalised atherosclerosis with a right sided subclavian occlusion and a narrowed right carotid causing the transient blindness in the right eye and the loss of touch in the left hand.

He was investigated by an arch aortogram which revealed several interesting features. The innominate and left common carotid arteries arose as a common trunk from the aortic arch. The left internal carotid artery was blocked just beyond its origin. The left vertebral artery was completely occluded. The right common carotid artery was very narrow. There was a short block in the right subclavian artery of about 1 cm. in length. The right vertebral artery filled retrogradely and the distal part of the right subclavian artery filled from it and through an anastomosis around the thyrocervical trunk.

It was felt that his most important symptom was cerebral, and it was decided to remove the partial occlusion from the right common carotid by endarterectomy.

On the 20th May, 1966, Mr. Terblanche exposed the common, internal and external carotid arteries. This was done using surface cooling hypothermia carried out by Professor Clutton-Brock. The patient was cooled to 29°C. Clamps were applied to the arteries and an incision was made into the internal carotid extending down into the common carotid. Atheroma involving the whole area was found. A soft platelet clot was found on the surface of the atheroma at the origin of the internal carotid. The stripping was technically difficult because of the extensive atheroma extending up and down the carotid vessels.

When the clamps had been on for almost fifteen minutes a local by-pass of polythene tubing was inserted into the lower carotid over a length of about 7 cms. Some atheromatous material was also removed from the origin of the external carotid artery. There was now a good back flow from both internal and external carotid arteries. Both the upper flap of atheroma in the internal carotid and the lower flap of atheroma in the external carotid were sutured down with interrupted silk sutures. Closure of the artery was performed from above downwards with continuous black silk leaving the by-pass *in situ* until near the end of the anastomosis.

When the clamps were removed there was a good flow and pulsation in the arteries. The surface incision was closed with silk, catgut being used deep to it. The patient was rewarmed and his immediate post-operative course was quite satisfactory.

However, late that evening he developed sudden cerebral signs and symptoms. He was noticed to be lethargic and speechless. He was unable to respond to simple orders but reacted to painful stimuli. On examination there was generalised hype-tonicity but no localising signs of central nervous system lesions. The pupils were equal and reacted to light. The fundi were normal.

His wound was satisfactory and the pulse rate, blood pressure and respiratory rate were within normal limits. The temperature was 100°F. Ankle clonus was elicited on both sides. A diagnosis of cerebral œdema was made and the possibility of a cerebro-vascular accident was considered.

The following morning (21st May) ankle clonus could be elicited on the left side but no longer on the right. The carotid artery was re-explored by Mr. Peacock and Mr. Terblanche, again under surface cooling hypothermia.

Both the internal and external carotids had retrograde pulsation. There was a region of thrombosis in the common carotid right up to the bifurcation. It was felt that a slight indentation produced by the interrupted sutures used to hold down the proximal flap of atheroma had caused turbulence resulting in thrombosis.

A saphenous vein graft was removed from the right groin. The internal carotid was transected above the suture line. The distal clot was sucked out with great care and a good back flow was obtained. An end-to-end anastomosis of the upper end with the graft was made. The internal carotid was tied off at the bifurcation and all the blood clot from the common and external carotid was cleared until there was a good back flow and pulsation from the distal external carotid. The vein by-pass was anastomised end-to-side of the common carotid. There was now a good flow and pulsation in both the external and internal carotid arteries.

In the post-operative period his condition never really improved and he was kept in the intensive care unit. He was anticoagulated intravenously with heparin. The rate of flow of heparin was regulated according to clotting time results which were done every six hours.

By the day after the operation he had a marked left flaccid hemiplegia. When seen on the second post-operative day by a neurologist he had a flaccid quadriplegia with bilateral extensor plantars and generalised hypotonia. The pupils were fixed and dilated. The femoral vessels revealed severe sclerotic change. There was no swallowing or cough reflex but no other dramatic demonstration of brain-stem nuclear lesion. Nevertheless it was felt that he had suffered brain-stem infarction as a result of basilar insufficiency and that no further procedure would prove useful. The demonstration of basilar filling from the occipital artery on previous arteriograms tended to suggest how precarious the brain-stem blood supply must have been.

The patient died that afternoon, the 23rd May, 1966.

Post-mortem revealed severe generalised atheroma and an old myocardial infarct. The coronary arteries were open but severely affected by atheroma. Both the renal arteries were normal. The mouths of the innominate, left common carotid and left sub-clavian arteries were open. The right subclavian was almost occluded by a plaque of atheroma at its origin. Both vertebral arteries were open. The left common carotid showed a patch of calcified atheroma just before the bifurcation. The external carotid was normal. The internal carotid was completely occluded by old thrombus one centimetre from the origin. The saphenous vein bypass graft had been working adequately.

The brain was heavy and the convolutions were flattened. The cerebellar tonsils had herniated through the foramen magnum. The circle of Willis showed mild atheroma only. Both middle cerebral arteries showed considerable atheroma, more marked on the right than on the left. After fixation and serial section of the brain, many small hæmorrhagic infarcts, up to three centimetres in diameter, were seen scattered throughout the cerebral hemispheres.

### DISCUSSION

Three factors usually account for the cerebro-vascular insufficiency symptoms in patients with occlusive disease of the extracranial cerebral arteries. These are a decrease in forward flow, a deficiency in collateral supply to the brain, and embolisation from ulcerated atheromatous lesions. The severity of the symptoms depends on the multiplicity of the occlusive sites and the adequacy of the collateral network.

It is estimated that at least 40% of cerebro-vascular accidents are due to extracranial vascular disease. Hutchinson and Yates (1961) made a detailed autopsy study of a hundred patients who had died with a clinical diagnosis of cerebral thrombosis. 14 had complete thrombosis of an intracranial artery, six of which were extensions from extra-cranial thrombosis. 58 had significant disease of an external cerebral artery, of which 18 were carotid only, 7 were vertebral only and 33 were carotid and vertebral. The remaining 28 had a wrong initial diagnosis and were mostly cerebral hæmorrhage or intracranial tumour.

The brain is supplied by four main vessels, the two internal carotid and the two vertebral arteries. When one internal carotid artery is occluded the external carotid becomes an important collateral vessel, blood from it reaching the circle of Willis mainly by the ophthalmic artery.

The patient had a very significant though not complete occlusion of his right internal carotid in 1964. He was reported to have an excellent collateral supply *via* the occipital artery to the basilar. It will be remembered that an anastomosis normally exists between the descending branch of the occipital, the deep cervical branch of the costocervical (branch of subclavian), and the vertebral which eventually forms the basilar.

The intracranial circulation rate was retarded by stenosis of the right internal carotid. It was still more retarded because the right external carotid which, as mentioned above, becomes an important collateral vessel) had to supply to basilar *via* the occipital. Also the right vertebral was non-functional, because the right sub-clavian was occluded.

The arch aortogram done just before the first operation showed that the right vertebral artery filled retrogradely and the distal part of the right subclavian artery filled from it. This is the ' subclavian steal ' syndrome and may occur in cases of occlusion of the innominate artery or the first part of the subclavian artery with reversal of blood flow in the vertebral artery. Such patients may present with episodes of vertebro-basilar insufficiency. When the arm on that side is exercised the vertebral artery acts as a collateral vessel to that arm. This reduces the blood reaching the brain from the circle of Willis and results in transient cerebral symptoms. The commonest symptoms are episodic vertigo, disorders of vision and ' dropping attacks ' in which the patient falls to the ground but remains conscious. Other symptoms include paresis of 1-4 limbs, transient numbness and paræsthesiæ of the limbs, intense occipital headache and buzzing in the ears. The patient presented several of these and indeed was considered to have suffered brain-stem infarction as a result of basilar insufficiency.

On clinical grounds alone it is often very difficult to diagnose whether a patient's symptoms are due to an occlusion of the internal carotid artery. The typical symptoms are blindness of the ipsilateral eye and contralateral hemiplegia with aphasia if the dominant hemisphere is involved. These may be transient in which case the likely cause is arterial stenosis. However, the typical clinical syndrome of internal carotid occlusion is not often seen. In a series of 200 patients with occlusion of the internal carotid artery, presenting symptoms included unconsciousness, headache, vomiting, bruit audible to patient, deafness, convulsions, dysphagia and severe mental changes apart from the more common motor and sensory symptoms, visual disturbances and speech difficulties (Rob, 1965).

Clinically two types of occlusion of the internal carotid are seen; stenosis or partial occlusion, and complete occlusion. It is often impossible by current techniques to restore blood flow in complete occlusion if the clot has spread into the cranial part of the carotid or vertebral systems. Here it soon becomes adherent and cannot be extracted. It is essential to obtain a satisfactory retrograde flow from a patent distal arterial tree if an arterial reconstruction procedure is to succeed.

Stenosis on the other hand is easy to deal with surgically. No matter how severe the patient's generalised disease is, it is normal for the internal carotid to be unaffected from the stenosis to the circle of Willis. Thus the immediate outflow tract is a good vessel and surgery is of value.

In a study of 100 patients who had had a major hemiplegia, it was found that in more than two-thirds there was a history of previous transient episodes before the major catastrophe (Rob, 1965). The transient symptoms are usually caused by a partial occlusion. This emphasizes the need for early surgical measures.

In a series of 407 patients with occlusive lesions of the extracranial arteries, surgical treatment was employed in 372 in whom the blood flow to the brain was reduced significantly (De Bakey, 1961). They were treated by endarterectomy and in many this was combined with patch graft angioplasty. Both the immediate and long term results of operation were extremely gratifying. Normal circulation was restored in all patients with occlusions involving the great vessels arising from the aortic arch and in 96% of patients with incomplete occlusive lesions involving the internal carotid and vertebral arteries.

It may be mentioned in passing that surgical procedures designed to increase the efficiency of the collateral circulation in the brain are rarely performed today. These included the formation of a carotid artery to jugular vein anastomosis, revascularization of the ischæmic part of the brain by a collateral circulation developed from a graft of temporal muscle implanted in the cerebral cortex, and cervical and perivascular sympathectomy. Most of these were shown by De Souza Pereira to be of little value (De Souza Pereira, 1955).

At post mortem, complete occlusion of the patient's *left* internal carotid was found. The saphenous vein by-pass graft was working adequately. It must be presumed that the patient's episode on the evening after the first operation must have been due to an occlusion of his left carotid and that this was the reason for the failure of both the first and the second operations on the right side. The fact that the left vertebral was not shown on the x-ray may be due to a fault in x-ray technique. It was found to be patent at post-mortem.

Vascular surgical techniques have advanced considerably and with careful assessment of the indications for surgery, the mortality and morbidity of operation can kept at very low levels. The degree of residual neurological disability seems to be improved in a fair proportion of surgically treated cases. Most important of all, prophylactic surgery is of value since very little can be done for the patient who has had a major cerebro-vascular accident.

### REFERENCES

(1) Cole, D. S. (1965) "Surgery in extracranial cerebro-vascular disease". *Brit. J. Surg.*, **52,** 892.

(2) D Bakey, M. E., Crawford, E. S., Morris, G. C., Jun., and Cooley, D. A. (1961) "Surgical considerations of occlusive disease of the Innominate, Carotid, Subclavian and Vertebral Arteries". *Ann. Surg.*, **154**, 698.

(3) De Sousa Pereira, A. (1955) "Surgical treatment of Internal Carotid Thrombosis". *Ann. Surg.*, **141**, 218.

(4) Hutchison, E. C., and Yates, P. O. (1961) " Cerebral Infarction : The role of stenosis of Extracranial Arteries ". M.R.C. Report No. 300, London; H.M. Stationery Office.

(5) Rob., C. G. (1965) " Diseases of the extracranial cerebral arteries ". *Clinical Surg.*, **9,** 134. London; Butterworth.

(6) Roberts, W. M. (1966) " The place of surgery in the prevention and treatment of stroke caused by lesions of the extracranial arteries ". *S. Afr. Med. J.*, **40,** 251.

## Figures and Tables

**Plate XXVII f1:**
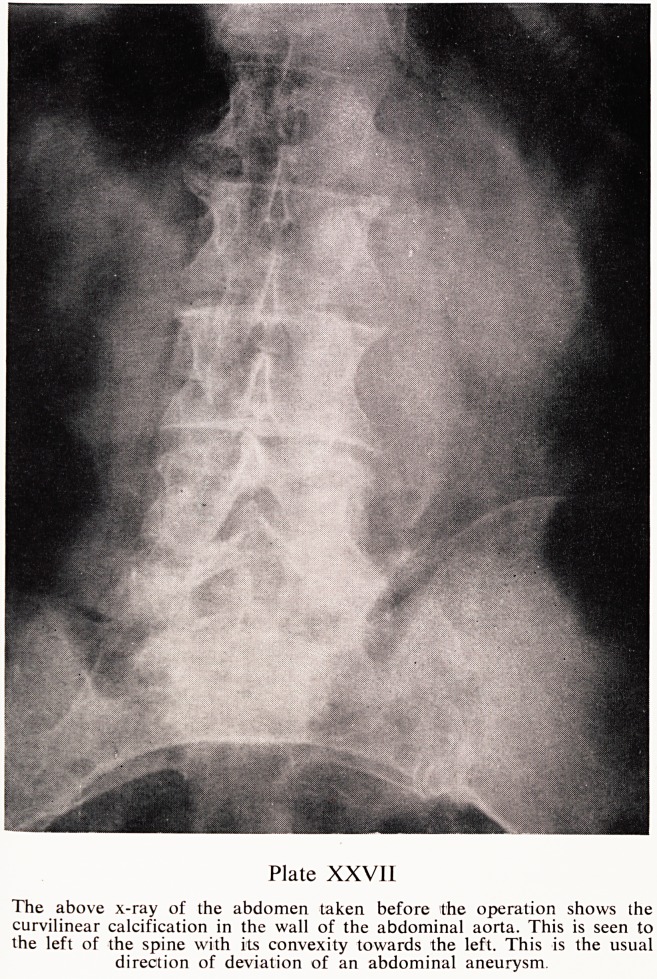


**Plate XXVIII f2:**
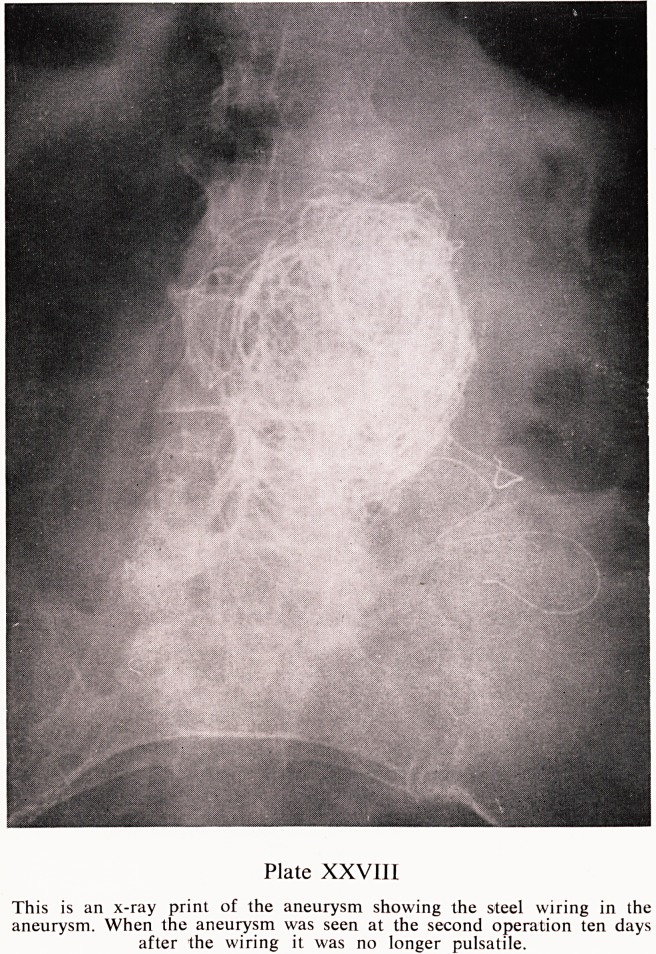


**Figure f3:**
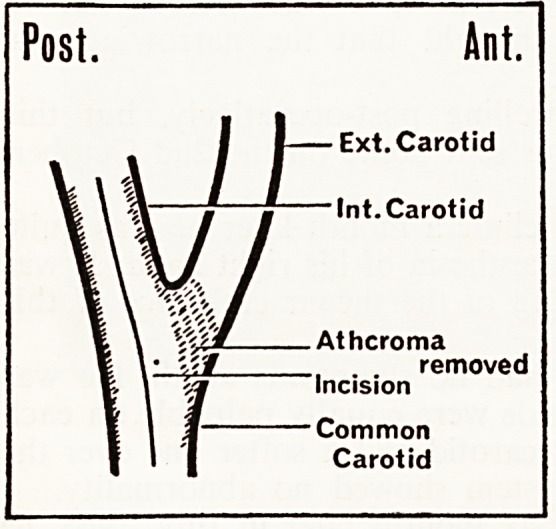


**Figure f4:**